# Acetylshikonin from Zicao Prevents Obesity in Rats on a High-Fat Diet by Inhibiting Lipid Accumulation and Inducing Lipolysis

**DOI:** 10.1371/journal.pone.0146884

**Published:** 2016-01-15

**Authors:** Meiling Su, Wendong Huang, Banghao Zhu

**Affiliations:** Department of Pharmacology, Cardiac and Cerebral Vascular Research Center, Zhongshan School of Medicine, Sun Yat-sen University, Guangzhou, China; Bambino Gesù Children's Hospital, ITALY

## Abstract

Various drugs have been developed to treat obesity, but these have undesirable secondary effects, and an efficient but non-toxic anti-obesity drug from natural sources is desired. This study investigated the anti-obesity effects and mechanisms of action of acetylshikonin (AS)—which is used in traditional Chinese medicine—in rats on a high-fat diet (HFD). Rats were fed a normal diet or an HFD; the latter group was received no treatment or were treated with 100, 300, or 900 mg/kg AS extract by intragastric administration for 6 weeks. In addition, 3T3-L1 adipocytes were treated with AS and the effects on adipogenesis and lipolysis were evaluated by western blot analysis of adipogenic transcription factors and lipid-metabolizing enzyme levels and the phosphorylation status of protein kinase (PK) A and hormone-sensitive lipase (HSL). AS prevented HFD-induced obesity including reduction in body weight, white adipose tissue content, liver mass, and serum triglyceride and free fatty acid levels in rats. It also suppressed the expression of adipogenic differentiation transcription factors and decreased the expression of the adipocyte-specific proteins HSL and adipose triglyceride lipase (ATGL). Furthermore, AS treatment induced lipolysis, leading to the release of glycerol and increased in PKA and HSL phosphorylation. These findings demonstrate that AS has anti-obesity effects in a rat model and may be a safe treatment for obesity in humans.

## Introduction

Obesity has become a global epidemic, especially in developed countries. In 2013–2014, More than 42 million children under the age of 5 and more than 1.9 billion adults 18 years and older were overweight [1]. Obesity is a complex metabolic disorder linked to an increased risk of common and serious human diseases, including type 2 diabetes mellitus, coronary atherosclerotic heart disease, and cardiovascular diseases such as hypertension and hyperlipidemia [2, 3]. Obesity is defined as excessive fat mass and expansion of adipose tissue resulting from adipocyte hypertrophy and hyperplasia that lead to weight gain and a body mass index > 30 kg/m^2^. The main cause of obesity is an imbalance between energy intake and expenditure [4]. White adipose tissue plays a critical role in the regulation of energy homeostasis by secreting adipokines (e.g., leptin, adiponectin, and resistin), which have been linked to obesity and metabolic disorders [5, 6]. The major contribution of white adipose tissue to whole-body energy metabolism is through lipolysis, in which stored triglycerides (TGs) are released as non-esterified free fatty acid (NEFA) and glycerol [7]. This process is regulated by cyclic (c) AMP, which induces the activation of PKA, which in turn activates downstream effectors that trigger TG lipolysis [8].

Various anti-obesity drugs including orlistat, lorcaserin, and extended-release phentermine/topiramate and naltrexone/bupropion have been approved by the U.S. Food and Drug Administration for chronic weight management in obese adults [9]. Although clinically effective, these drugs have side effects such as gastrointestinal disease, asthenia, mental disturbance, or cardiovascular effects [10]. Therefore, an efficient but non-toxic anti-obesity drug from natural sources is desired. Acetylshikonin (AS) and its extract are both derived from the traditional Chinese medicine Zicao at purities of 98.4% and > 80%, respectively, as detected by high performance liquid chromatography. Zicao is a broad-spectrum compound with anti-inflammatory, antitumorigenic, antibacterial, antiviral, and wound-healing properties [11, 12, 13, 14]. Zicao and shikonin have both been used to treat obesity [15, 16, 17]; the latter has been proposed to inhibit fat accumulation in 3T3-L1 adipocytes [18].

Existing anti-obesity drugs function by inducing adipocyte apoptosis, inhibiting lipid accumulation, and stimulating lipolysis [19] by targeting a variety of molecules. Peroxisome proliferator-activated receptor (PPAR) γ and CCAAT/enhancer-binding protein (C/EBP) α are key transcription factors during adipocyte differentiation [20]; shikonin has been shown to prevent lipid accumulation by inhibiting their expression [16, 18]. Lipid-metabolizing enzymes such as hormone-sensitive lipase (HSL) and adipose TG lipase (ATGL) are key regulators of lipolysis that act by hydrolyzing intracellular triacylglycerol and diacylglycerol to release NEFA and glycerol [21]. HSL phosphorylation is mediated by the PKA signaling cascade, and its activation increases TG lipolysis in adipocytes [22]. ATGL shows high substrate specificity for triacylglycerol and very low affinity for diacylglycerol in lipolysis [23]. Hence, HSL, ATGL, and perilipin are essential for catecholamine-stimulated lipolysis [24, 25]. However, it remains unclear whether AS has similar effects on lipid metabolism and hence, on obesity. The present study addressed this issue by evaluating the anti-obesity effect of AS and the associated mechanisms in a rat model of obesity induced by a high-fat diet (HFD).

## Results

### AS Extract Reduces HFD-Induced Obesity in Rats

**Fig 1:**
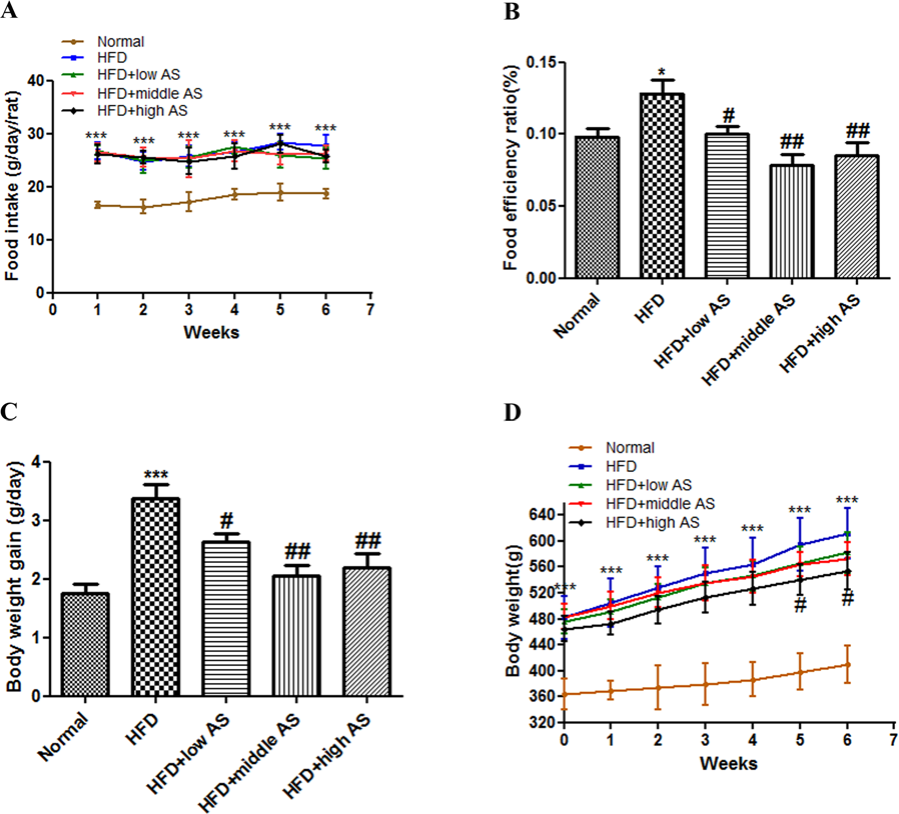
AS extract reduces HFD-induced obesity in rats. Rats were randomly divided into five groups: normal control, HFD model, and HFD with AS extract (100, 300, or 900 mg/kg). The extract was intragastric administered for 6 weeks. (**A**) Food intake per rat was recorded six times a week. (**B**) Food efficiency ratio was calculated as body weight gain divided by food intake. (**C**) Change in body weight was measured six times a week. (**D**) Body weight gain measured in a given week was subtracted from the weight in the previous week. Values are expressed as mean ± SE (n = 30). Significant differences were observed between normal and HFD groups. *P < 0.05, **P < 0.01, ***P < 0.001 vs. normal group; ^#^P < 0.05, ^##^P < 0.01, ^###^P < 0.001 vs. model group.

Rats were divided into five groups: normal diet (normal control); HFD without treatment (HFD model); and HFD with AS extract treatment at 100, 300, and 900 mg/kg (low, middle, and high doses, respectively). The food efficiency ratio and body weight of the HFD model group were increased by 30.6% and 49.3%, respectively, relative to the normal controls (P < 0.05 and P < 0.01, respectively) (Fig 1). Weekly food intake per rat did not differ among HFD groups during the experimental period (P > 0.05) (Fig 1A); however, food efficiency ratio was lower in AS extract-treated rats [10.0% ± 0.14% (low dose), 7.8% ± 0.18% (middle dose), and 8.5% ± 0.23% (high dose) vs. 12.8% ± 0.23% (HFD model); P < 0.05, P < 0.01, and P< 0.01, respectively] (Fig 1B). Body weight gain was also reduced in AS extract-treated as compared to untreated HFD rats (P < 0.05) (Fig 1D). None of the rats exhibited any pathological signs during the experimental period.

### AS Extract Reduces Adipose Tissue Weight, White Adipocyte Size and Liver Accumulation in Obese Rats

**Fig 2:**
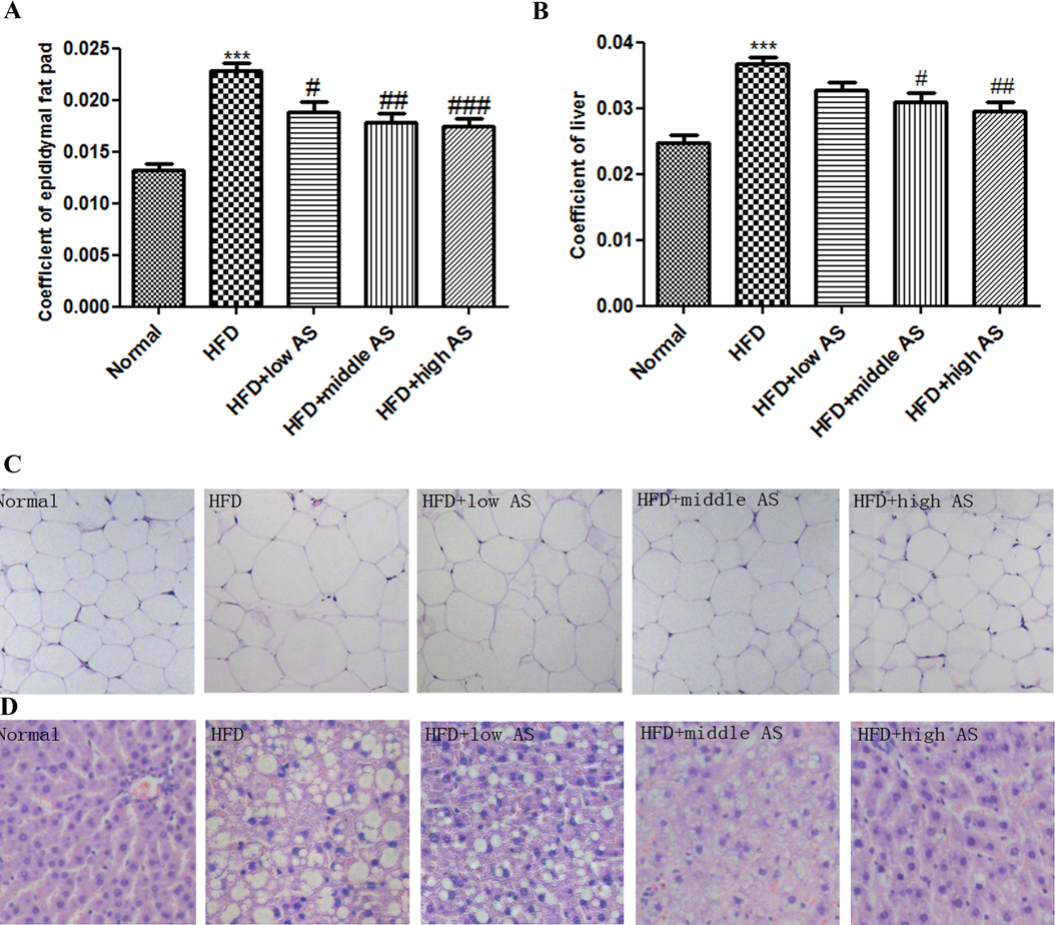
AS extract reduces adipose tissue weight, white adipocyte size and liver accumulation in obese rats. Coefficients of (**A**) epididymal adipose tissue and (**B**) liver were calculated after rats had fasted for 12 h at the end of experiment according to the following equation: coefficient = tissue (g/rat)/weight (g/rat). (**C**) Epididymal adipose tissue and (**D**) liver were stained with hematoxylin and eosin for histopathological examination. Images were acquired at 400× magnification on a light microscopy. Values are expressed as mean ± SE (n = 6). Significant differences were observed between normal and HFD groups. *P < 0.05, **P < 0.01, ***P < 0.001 vs. normal group; ^#^P < 0.05, ^##^P < 0.01, ^###^P < 0.001 vs. model group.

The epididymal adipose tissue coefficient was lower in rats on an HFD treated with l00 mg/kg (18.7% ± 0.23%), 300 mg/kg (18.0% ± 0.23%), or 900 mg/kg (17.9% ± 0.16%) AS extract than in the HFD model group (22.8% ± 0.19%) (Fig 2A and 2B). A histological analysis revealed larger adipocytes in the HFD model as compared to normal controls; however, AS extract treatment reduced adipocyte size as compared to untreated HFD model rats (P < 0.001) (Fig 2C). In addition, the liver of HFD rats showed typical features of fatty liver such as lipid droplet infiltration (Fig 2D); this was reversed by AS extract treatment. These results suggest that AS extract reduces adipose tissue mass by preventing fat accumulation in the liver of obese rats.

### AS Extract Decreases Serum Free Fatty Acid (FFA) and TG Levels in Obese Rats

Blood lipid levels were higher in HFD than in normal control rats (Table 1). Meanwhile, serum FFA levels were reduced by approximately 32.2% and 35.5% in HFD rats treated with 300 and 900 mg/kg AS extract, respectively, relative to HFD model rats (P < 0.05 and P < 0.01, respectively; n = 6). Similarly, serum TG levels were reduced by approximately 22.9% and 39.9% in HFD rats treated with 300 and 900 mg/kg AS extract, respectively, as compared to HFD rats without treatment (P < 0.05 and P < 0.01, respectively; n = 6).

**Table 1:** Effect of acetylshikonin on biochemical parameters of serum in HFD induced obese rats.

	Normal	HFD	HFD+low AS	HFD+middle AS	HFD+high AS
**TC (mM)**	1.24±0.10	1.68±0.33*	1.48±0.28	1.38±0.25	1.64±0.28
**LDL (mM)**	0.25±0.03	0.58±0.19**	0.51±0.12	0.41±0.18	0.49±0.14
**HDL (mM)**	0.65±0.03	0.48±0.09**	0.45±0.09	0.45±0.09	0.51±0.08
**TG (mM)**	0.79±0.17	1.21±0.10^***^	1.11±0.32	0.93±0.29^#^	0.73±0.27^##^
**FFA (mM)**	0.43±0.27	0.70±0.32**	0.62±0.29	0.51±0.41^#^	0.49±0.12^##^

TC, total cholesterol; LDL, low density lipoprotein cholesterol; HDL, high density lipoprotein cholesterol; TG, triglyceride; FFA, free fatty acid. Values are expressed as mean ± SE of 6 rats.

*P < 0.05

**P < 0.01

***P < 0.001 vs. normal group

^#^P < 0.05

^##^P < 0.01

^###^P < 0.001 vs. model group, significantly different from the HFD rats.

### AS Inhibits Differentiation and Fat Accumulation in Preadipocyte Cells

**Fig 3:**
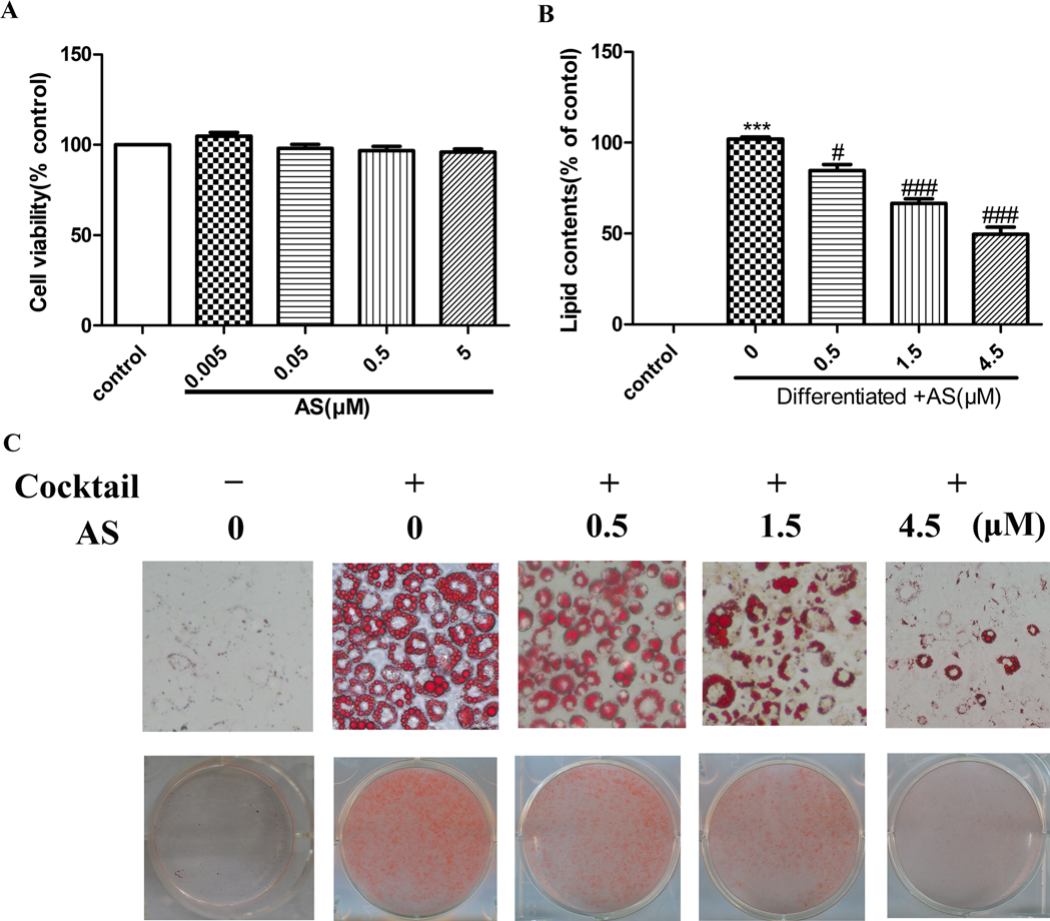
AS inhibits 3T3-L1 preadipocyte differentiation and fat accumulation. (**A**) Cell viability was assessed with the MTT assay24 h after AS treatment. (**B**) Relative lipid content. (**C**) Inhibition of fat droplet formation by AS. 3T3-L1 cells were seeded and induced to differentiate for 8 days with or without AS in 6-well plates, then stained with Oil Red O and imaged by light microscopy (200×). Values are expressed as mean ± SEM. Significant differences were observed between the untreated and differentiated control cells. *P < 0.05, **P < 0.01, **P < 0.001 vs. AS-treated cells; ^#^P < 0.05, ^##^P < 0.01, ^###^P < 0.001 vs. differentiated control cells.

The viability of 3T3-L1 cells incubated with various concentrations of AS for 24 h was evaluated with the 3-(4, 5 -dimethylthiazol-2-yl)-2, 5-idiphenyl tetrazolium bromide (MTT) assay. Treatment with AS at concentrations ranging from 0.005 to 5 μM had no effect on viability (P > 0.05; n = 6) (Fig 3A). Staining of fully differentiated adipocytes with Oil Red O revealed that adipocyte differentiation and lipid accumulation were suppressed in a dose-dependent manner in the presence of AS (P < 0.05) (Fig 3B and 3C).

### AS Inhibits the Expression of Adipogenic Transcription Factors and Lipid-Metabolizing Enzymes

**Fig 4:**
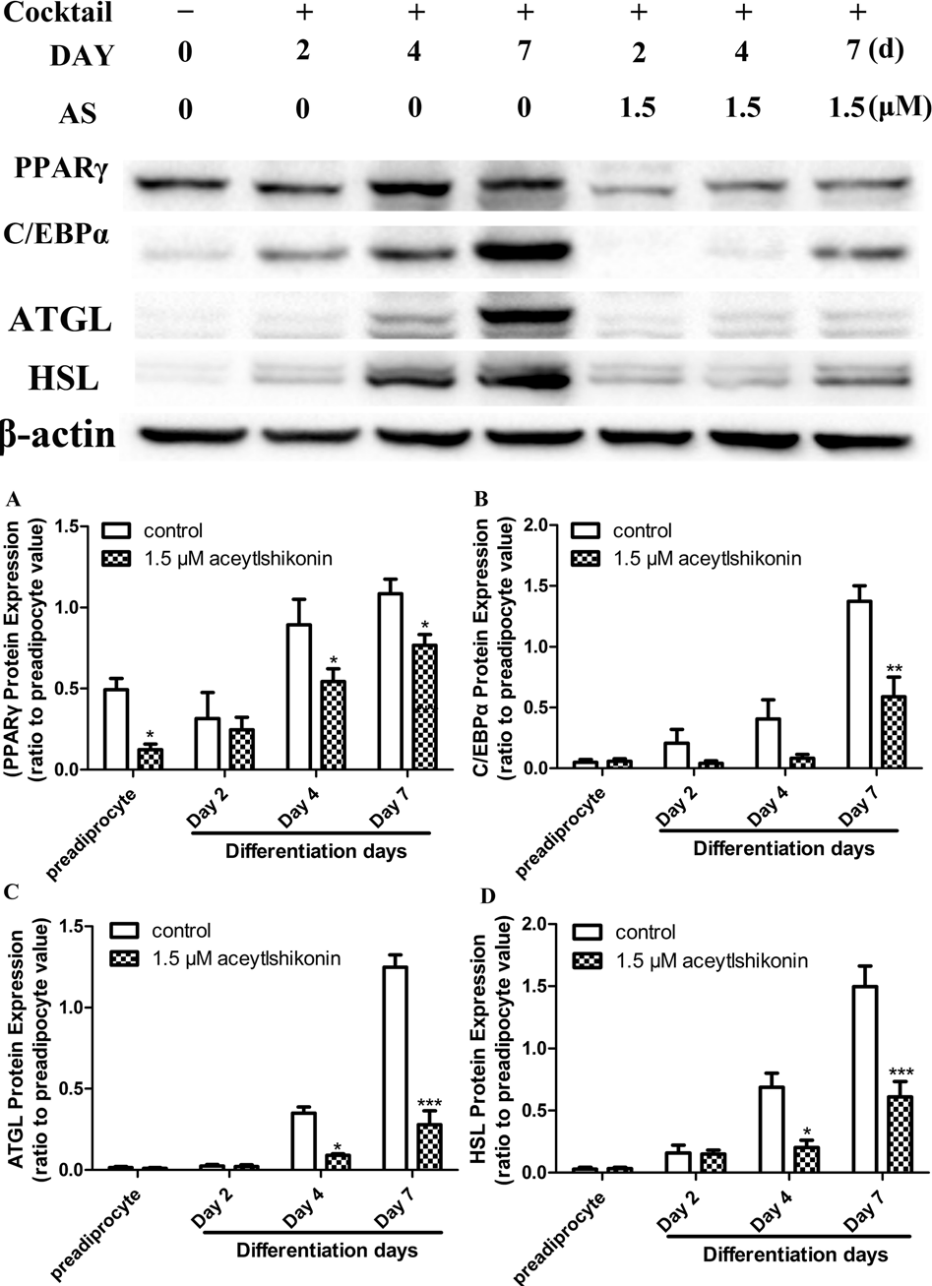
AS inhibits PPARγ, C/EBPα, HSL, and ATGL expression in adipocytes. Protein levels were assessed by western blotting in 3T3-L1 cells cultured for 0, 2, 4, and 7 days in the presence or absence of 1.5 μM AS during adipogenesis. Immunoblots are representative of five independent experiments; β-actin served as a loading control. *P < 0.05, **P < 0.01, ***P < 0.001 vs. untreated adipocytes at the same time point.

PPARγ and C/EBPα protein levels were markedly reduced by AS (1.5 μM) treatment relative to untreated cells during adipogenesis. Similarly, the expression of the adipogenic lipid-metabolizing enzymes HSL and ATGL was reduced in the presence of 1.5 μM AS (P < 0.05; n = 5) (Fig 4). These data indicate that AS inhibits adipocyte differentiation while also modulating lipid metabolism in mature adipocytes.

### AS Induces Lipolysis in Mature Adipocytes

**Fig 5:**
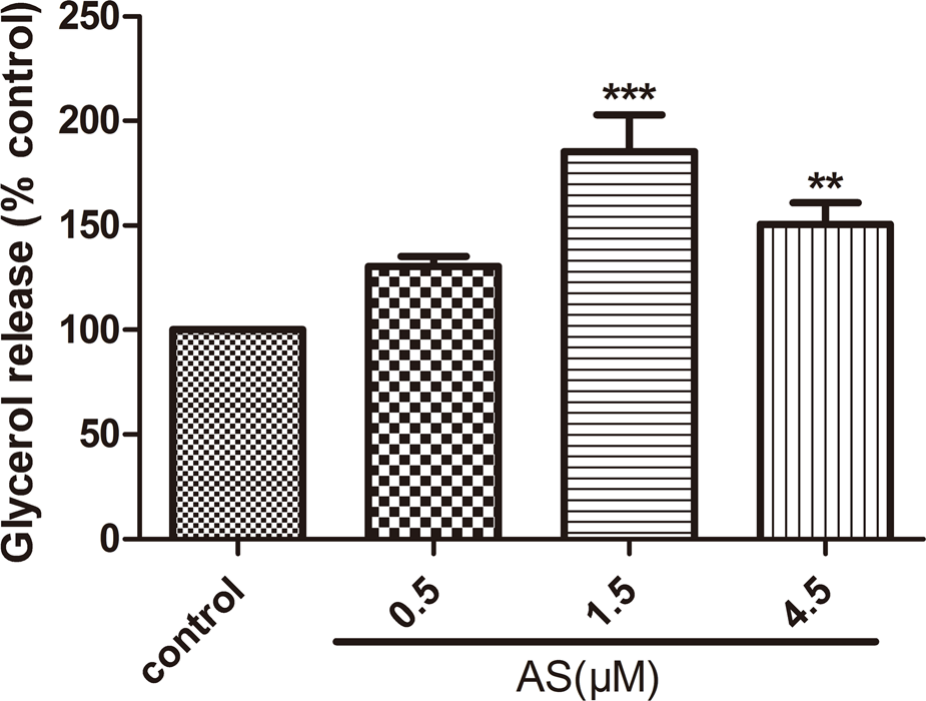
AS induces lipolysis in mature adipocytes. Mature 3T3-L1 cells were incubated with various concentrations of AS (0, 0.5, 1.5, and 4.5 μM) for 24 h. Values are expressed as mean amount (± SE) of glycerol released per well as a percentage of the control value. Four replicates were carried out for each treatment. Means denoted with a different letter are significantly different (P < 0.05).

To determine whether AS affects lipolysis, fully differentiated 3T3-L1 adipocytes were serum-deprived and then incubated with various concentrations of AS (0, 0.5, 1.5, and 4.5 μM) for 24 h. At 1.5 and 4.5 μM, AS stimulated lipolysis and glycerol secretion, which was 0.85- and 0.50-fold higher, respectively, than in control cells (P < 0.01 and P < 0.05, respectively; n = 4) (Fig 5). AS had no effect on lipolysis at doses < 0.5 μM (data not shown).

### AS Induces Lipolysis in Mature Adipocytes by Activating the Phosphorylation and Activity of HSL and PKA

**Fig 6:**
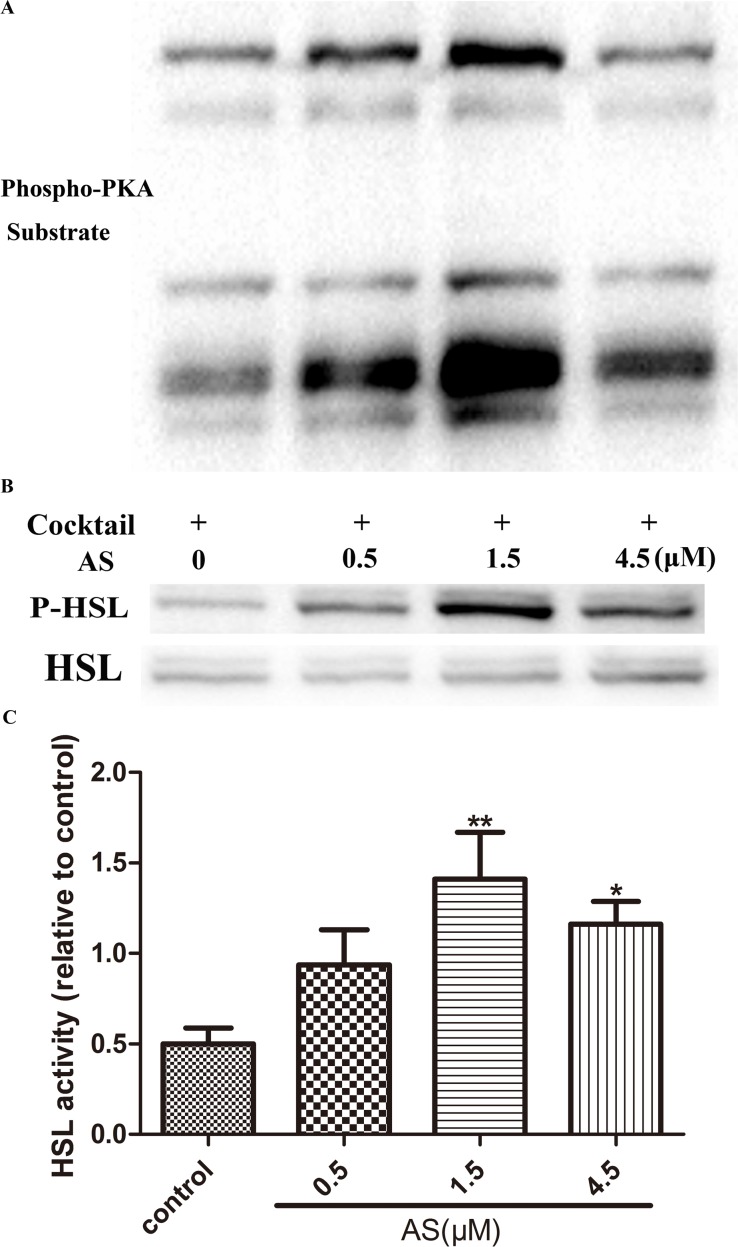
AS induces lipolysis in mature adipocytes by activating the phosphorylation and activity of PKA and HSL. Fully differentiated 3T3-L1 adipocytes were incubated in the absence and presence of various concentrations of AS (0, 0.5, 1.5, and 4.5 μM) for 3 h. Immunoblots are representative of seven independent experiments; β-actin served as a loading control. *P < 0.05, **P < 0.01, ***P < 0.001 vs. untreated adipocytes at the same time point.

We also examined the effect of AS on the phosphorylation of PKA and HSL. Fully differentiated 3T3-L1 adipocytes were incubated in the absence or presence of various concentrations of AS (0, 0.5, 1.5, and 4.5 μM) for 3 h. AS treatment increased the phosphorylation—and hence, the activity—of PKA relative to the control. A western blot analysis revealed an increase in the phosphorylation of HSL at Ser563 in AS-treated as compared to untreated adipocytes (P < 0.05; n = 7) (Fig 6).

## Discussion

Zicao, which is widely used in China to treat infectious and inflammatory diseases, contains important naphthoquinone derivatives including AS, shikonin, β-hydroxyisovaleryshikonin, β, β-dimethylacrylshikonin, deoxyshikonin, and isovalerylalkannin [26]. Many studies have demonstrated the antitumorigenic, anti-bacterial, anti-inflammatory, anti-viral, and wound-healing activities of Zicao [27, 28], and it was recently shown to inhibit lipid accumulation [17].

The present study investigated the anti-obesity effects of AS and its extract in rats with HFD-induced obesity and 3T3-L1 adipocytes. The intragastric administration of AS extract had no effect on food intake but markedly reduced food efficiency ratio, body weight gain, and adipose tissue weight. HFD model rats showed typical histological markers of obesity in the adipose tissue as well as fatty liver [29]. AS extract reduced adipocyte size and volume and inhibited hepatic lipid droplet accumulation, thereby preventing the development of fatty liver. Moreover, serum FFA and TG levels, which were elevated in HFD model rats, were decreased in a concentration-dependent manner by AS treatment. These results indicate that AS extract can improve the symptoms of obesity.

Adipocyte lipid accumulation can increase the size and/or number of adipocytes and lead to obesity [30]. However, inhibiting adipogenesis may not be a viable treatment for obesity given that limiting adipose tissue expansion can result in insulin resistance [31, 32]. Nonetheless, some herbal compounds have been used to inhibit adipogenesis and obesity induced by an HFD [33, 34, 35]. PPARγ and C/EBPα are two transcription factors that mediate terminal adipocyte differentiation [20, 36]. PPARγ induces the up-regulation of adipocyte-specific markers associated with lipid metabolism, such as HSL and ATGL [37]. We evaluated the effect of AS treatment on the expression of PPARγ, C/EBPα, HSL and ATGL in 3T3-L1 preadipocytes by western blotting. The levels of all four of these proteins were downregulated in the presence of 1.5 μM AS extract during adipogenesis, suggesting that AS inhibits the differentiation of preadipocytes by suppressing PPARγ and C/EBP expression, and induces the degradation of lipids stored in adipocytes by inhibiting HSL and ATGL expression.

Lipolysis may play an important role in regulating obesity and related disorders such as diabetes mellitus, fatty liver disease, and cardiovascular diseases. White adipose tissue regulates energy metabolism via lipolysis, which supplies energy to other tissues through breakdown of triacylglycerol and FA release [38]. Adipocyte stimulation leads to an increase in intracellular cAMP levels; the consequent activation of PKA activates the downstream molecules HSL, ATGL and perilipin, leading to tri- and diacylglycerol hydrolysis and the release of FFA and glycerol [39, 40]. We showed that AS treatment increased lipolysis and glycerol release in fully differentiated adipocytes by 0.85-fold over control cells. HSL induces lipolysis by hydrolyzing intracellular tri- and diacylglycerol, with the latter being broken down into FFA and glycerol by ATGL [41]. In addition, perilipin—which is located on the surface of adipocytes lipid droplet—is a key protein in lipolysis regulation [42] that is activated by PKA-mediated phosphorylation. Perilipin prevents lipolysis by acting as a barrier for HSL interaction [43] but upon phosphorylation by PKA, it triggers HSL phosphorylation on lipid droplets [41]. We demonstrated that AS treatment increases the phosphorylation and activity of HSL and PKA. Taken together, our results provide evidence that AS can reduce lipid levels by inducing triacylglycerol hydrolysis may via regulation of PKA signaling.

## Conclusions

The herbal compound AS exerts anti-obesity effects by inhibiting adipocyte lipid accumulation and inducing the lipolysis of mature adipocytes. Our findings suggest that AS is an efficient, non-toxic, and safe alternative to anti-obesity drugs that are currently on the market.

## Materials and Methods

### Reagents and Antibodies

Dulbecco’s Modified Eagle’s Medium (DMEM) was purchased from GIBCO (BRL Life Technologies, Grand Island, NY, USA). Zicao were purchased from Xinjiang in China. Isobutyl-3-methyl-xanthine (IBMX), dexamethasone (DEX), human recombinant insulin, the MTT assay kit, Oil Red O, and dimethyl sulfoxide (DMSO) were purchased from Sigma-Aldrich (St. Louis, MO, USA). Glycerol (E1002 Glycerol Assay kit; 100 μl) was purchased from Polygen (Beijing, China). Antibodies against phosphorylated forms of AMP-activated protein kinase, HSL (Ser563), and PKA and horseradish peroxidase (HRP)-conjugated anti-rabbit secondary antibody were purchased from Cell Signaling Technology (Beverley, MA, USA). Antibodies against PPARγ, C/EBPα, and HSL were obtained from Abcam (Cambridge, UK). All other reagents were purchased from Sigma-Aldrich unless otherwise specified.

### Animals and Induction of HFD Model

Animal experiments were approved by the Animal Research Center of Sun Yat-sen University. Male Sprague-Dawley rats (n = 30, 6 weeks old) were housed three per cage under pathogen-free conditions with constant temperature (18°C–22°C) and humidity (55%–60%) on a 12:12-h light/dark cycle. Rats were allowed to acclimate for 1 week prior to experiments and were supplied with fresh food and water daily. Animals were randomly divided into normal control (n = 6) and HFD model (n = 24) groups. After 8 weeks, rats on an HFD showed significant weight gain and > 3-fold increases in blood lipid levels, and were randomly assigned to one of four groups (n = 6 each): HFD model (untreated) and HFD treated with low (100 mg/kg), middle (300 mg/kg), or high (900 mg/kg) doses of AS. Normal control rats were fed a standard diet while the HFD consisted of 10% lard, 1.25% cholesterol, 0.5% bile salts, 10% egg yolk powder, and 78.75% standard diet. Vehicle (distilled water) or AS extract was intragastrically administered for 6 weeks to HFD rats. Body weight and food intake were recorded six times a week.

### Biochemical Analysis

Rats were anesthetized and sacrificed after a 12-h fast at the end of the experiment. Blood was collected from the abdominal vena cava and centrifuged at 3000 rpm for 15 min at room temperature. Serum triglyceride (TG), total cholesterol, high-density lipoprotein (HDL) cholesterol, low-density lipoprotein (LDL) cholesterol and free fatty acid (FFA) levels were measured with a Model 7180 automated biochemical analyzer (Hitachi, Tokyo, Japan).

### Histological Analysis

White adipose tissue (epididymis) and liver were dissected from rats and immediately weighed and fixed in 10% formalin solution for 1 day. After dehydration in ethanol, the tissue was embedded in paraffin and cut into sections at a thickness of 5 μm, which were stained with hematoxylin and eosin. Sections were imaged on an IX71 inverted light microscope (Olympus, Tokyo, Japan) at 200× magnification.

### Cell Culture and Differentiation

Mouse fibroblast 3T3-L1 preadipocytes were purchased from the American Type Culture Collection (Manassas, VA, USA) and cultured in high-glucose DMEM containing 10% calf serum, penicillin (100 U/ml), and streptomycin (100 μg/ml) in an atmosphere of 5% CO_2_ and at 37°C. The medium was changed three times a week. Cells were grown to confluence in 6-well plates and differentiation was induced by adding an adipogenic cocktail containing 0.5 mM IBMX, 0.5 mM DEX, and 10 mg/l insulin in high-glucose DMEM with 10% fetal bovine serum (FBS) for 2 days. The medium was then replaced with DMEM with 10% FBS and 10 mg/l insulin for 2 more days, after which the medium was replaced with DMEM containing 10% FBS every 2 days until about 95% of cultured preadipocytes had differentiated into mature adipocytes. To assess the effect of AS on adipocyte differentiation, confluent 3T3-L1 preadipocytes were treated with 0, 0.5, 1.5, or 4.5 μM AS in the presence of the adipogenic cocktail for 7 days.

### Cell Viability Assay

The viability of 3T3-L1 cells was assessed with the MTT assay as previously described [22]. Cells were grown in 96-well plates at a density of 2 × 10^4^ cells/well. After adhering overnight, the cells were stimulated with AS in various concentrations (0.005, 0.05, 0.5 or 5 in DMSO). The control group was treated with DMEM with 10% FBS and 0.1% DMSO. After incubation for 24h at 37°C in 5% CO_2_, cells were washed three times with phosphate-buffered saline (PBS). MTT reagent (10 μl of a 1 mg/ml solution) and medium (100 μl) were added to each well, followed by incubation in the dark at 37°C for 4h. Formazan crystals were solubilized with DMSO, and the absorbance at 570 nm was measured on an Elx-800 microplate reader (BioTek, Winooski, VT, USA).

### Oil Red O Staining and Cell Quantification

Oil Red O staining was carried out according to a previously described method [44]. 3T3-L1 cells were induced to differentiate as described above, washed three times with PBS, fixed with 10% formalin for 1 h, then stained with Oil Red O (0.5% in 60% isopropanol) for 30 min. Adipocytes were washed with 70% ethanol in water and then air-dried. Stained cells were visualized by light microscopy and imaged at 200× magnification. Oil Red O-stained lipids were dissolved with 100% isopropanol and absorbance at 490 nm was determined with a spectrophotometer.

### Lipolysis Assay

Fully differentiated adipocytes (i.e., day 8) were serum-deprived overnight in Phenol Red-free medium containing 2% bovine serum albumin and treated with AS (0, 0.5, 1.5, or 4.5 μM) or left untreated for 24 h. At the end of the experiment, 100 μl of medium was removed from each well and transferred to a 96-well plate for assessment of glycerol levels. The reagent from the assay kit was added to each well and incubated at 37°C for 10 min, and absorbance at 570 nm was read with a microplate reader.

### Western Blot Analysis

Total protein from cells was extracted with lysis buffer containing a protease and/or phosphatase inhibitor cocktail. Protein concentration was quantified with a bicinchoninic acid kit. Proteins were separated by 8% sodium dodecyl sulfate polyacrylamide gel electrophoresis and then transferred to a polyvinylidene difluoride membrane (Millipore, Billerica, MA, USA), which was blocked with 5% non-fat dry milk in Tris-buffered saline with Tween 20 (TBST; 20 mM Tris-HCl, 150 mM NaCl, 0.1% Tween 20, pH 7.5) for 1 h. The membrane was then incubated overnight at 4°C with primary antibodies against the following proteins: PPARγ, C/EBPα, and HSL (1:1000); ATGL, phospho-HSL (Ser563), and phospho-PKA (1:1000). After washing three times with TBST, membranes were incubated with HRP-conjugated goat anti-rabbit IgG (1:1000) for 1.5 h. β-Actin antibody (1:3000; Cell Signaling Technology) was used as a loading control. Proteins were detected by enhanced chemiluminescence (Beyotime, Shanghai, China).

### Statistical Analysis

Data analysis was carried out using Graph Pad Prism v.5.0 (GraphPad, La Jolla, CA, USA). Data are expressed as mean ± standard error of the mean. Differences between groups were assessed with the Student’s paired t test or one-way analysis of variance, followed by a post hoc comparison with the Bonferroni test. P values < 0.05 were considered statistically significant.
